# PIAS1 Is a GATA4 SUMO Ligase That Regulates GATA4-Dependent Intestinal Promoters Independent of SUMO Ligase Activity and GATA4 Sumoylation

**DOI:** 10.1371/journal.pone.0035717

**Published:** 2012-04-23

**Authors:** Narasimhaswamy S. Belaguli, Mao Zhang, Andres-Hernandez Garcia, David H. Berger

**Affiliations:** Michael E. DeBakey Department of Surgery, Baylor College of Medicine, Michael E. DeBakey VA Medical Center, Houston, Texas, United States of America; University of Hong Kong, Hong Kong

## Abstract

GATA4 confers cell type-specific gene expression on genes expressed in cardiovascular, gastro-intestinal, endocrine and neuronal tissues by interacting with various ubiquitous and cell-type-restricted transcriptional regulators. By using yeast two-hybrid screening approach, we have identified PIAS1 as an intestine-expressed GATA4 interacting protein. The physical interaction between GATA4 and PIAS1 was confirmed in mammalian cells by coimmunoprecipitation and two-hybrid analysis. The interacting domains were mapped to the second zinc finger and the adjacent C-terminal basic region of GATA4 and the RING finger and the adjoining C-terminal 60 amino acids of PIAS1. PIAS1 and GATA4 synergistically activated IFABP and SI promoters but not LPH promoters suggesting that PIAS1 differentially activates GATA4 targeted promoters. In primary murine enterocytes PIAS1 was recruited to the GATA4-regulated IFABP promoter. PIAS1 promoted SUMO-1 modification of GATA4 on lysine 366. However, sumoylation was not required for the nuclear localization and stability of GATA4. Further, neither GATA4 sumoylation nor the SUMO ligase activity of PIAS1 was required for coactivation of IFABP promoter by GATA4 and PIAS1. Together, our results demonstrate that PIAS1 is a SUMO ligase for GATA4 that differentially regulates GATA4 transcriptional activity independent of SUMO ligase activity and GATA4 sumoylation.

## Introduction

GATA factors are zinc finger-containing transcription factors that play an important role in developmental processes, tissue differentiation and cell-type specific gene expression. Based on sequence similarity and expression pattern, GATA factors are grouped into 2 subgroups: GATA1/2/3 are mostly expressed in hematopoietic tissues and GATA4/5/6 are expressed in mesodermally- and endodermally-derived tissues such as, heart, vasculature, lungs, liver, intestines, gonads and various endocrine glands [1]. In the intestine GATA4 is expressed in a rostro-caudal gradient with a strongest expression in the duodenum and the jejunum and decreasing expression along the length of ileum and undetectable in colon [2]–[4]. GATA4 also exhibits a gradient expression along the crypt-villus axis [2], [3], [5]–[7]. Strong GATA4 expression is detected in terminally differentiated cells at the villus tip and in differentiating cells along the sides of the villi suggesting that GATA4 expression is associated with enterocyte differentiation.

In support of the role of GATA4 in enterocyte differentiation, GATA4 binding sites are present in the regulatory regions of several enterocyte expressed genes such as, lactase-phlorizin hydrolase (LPH) [8], sucrose isomaltase (SI) [6], intestinal fatty acid binding protein (IFABP/FABP-2) [5], [7], liver type fatty acid binding protein (LFABP/FABP-1) [9], claudin-2 [10], intestinal alkaline phosphatase (IAP) [5]. GATA4 binds to these sites and GATA4 binding has been shown to be essential for the expression of promoters of these differentiation marker genes. In intestine-specific GATA4 knockout animals the expression of FABP-1, LPH and various genes characteristic of jejunal epithelial transcriptome were downregulated in jejunum confirming the obligatory role of GATA4 in gut epithelial gene expression [2], [3]. Interestingly, several ileal epithelium-specific genes including apical sodium-dependent bile acid transporter (ASBT) and ileal lipid binding protein (ILBP), were upregulated in the jejunal epithelium in these animals suggesting that GATA4 plays a pivotal role in establishing the small intestinal segment identity by promoting jejunal-specific gene program while simultaneously repressing ileal-specific-gene program [2], [3].

GATA4 plays a central role in tissue-specific gene expression in various other tissue types such as, heart, gonads, and neuroendocrine tissues [1], [11]–[14]. Studies examining the mechanisms by which GATA4 contributes to tissue specific-gene expression in different tissue types have established that the ability of GATA4 to combinatorially interact with various ubiquitous and tissue-restricted factors is the basis by which GATA4 drives tissue- and cell type-specific gene program. GATA4 has been shown to physically and/or functionally interact with several GI tissue-expressed factors such as HNF-1α [6], [9], [15], [16], HNF4 alpha [17], Fog1/2 [18]–[20], GATA5 [21], Cdx-2 [6], [22] and the TGFβ signal transducing Smads [5] to regulate gene expression in GI tissues. In this study we sought to identify additional GATA4 interacting proteins expressed in the GI tissue using the yeast two-hybrid system. We have identified protein inhibitor of activated STAT1 (signal transducer and activator of transcription 1) [PIAS1], a protein with small ubiquitin related modifier (SUMO) ligase activity, as a small intestine-expressed GATA4 interacting protein and show that PIAS1 physically interacts with GATA4 and synergistically enhances GATA4 transcriptional activity on intestinal gene promoters such as IFABP and SI but not LPH. Further, we show that PIAS1 promotes GATA4 sumoylation on lysine 366 in agreement with a previous report [23]. However, in contrast to this previous report we show that in intestinal epithelial cells nuclear localization and transcriptional activity of GATA4 are independent of sumoylation and neither PIAS1 SUMO ligase activity nor GATA4 sumoylation are required for coactivation of intestinal epithelium expressed IFABP promoter.

## Materials and Methods

### Ethics statement

Animal experiments described herein were approved by the Baylor College of Medicine institutional animal care and use committee (protocol number AN-2825).

### Plasmids

Wild type and −40 GATA site mutated IFABP-luciferase reporter, and wild type and deletion mutants of GATA4 have been reported earlier [5]. Lactase phlorizin hydrolase (LPH)-luciferase reporter was constructed by cloning PCR generated −399 to +14 region of human LPH gene into KpnI-BglII sites of pGL2 basic vector. Sucrase isomaltase (SI)-luciferase reporter was similarly generated by cloning −342 to +30 fragment into MluI-XhoI sites of pGL2 basic vector. The murine PIAS1 expression vector, pCMVPIAS1, was a kind gift from K. Shuai [24]. FLAG tagged PIAS1 and deletion mutants of PIAS1 expressing different domains of PIAS1 (1–150, 1–480, 121–480, 300–480, 300–650 and 450 to 650) were constructed using PCR in the vector pCMVTag2C. The mammalian two-hybrid Gal4 DNA binding domain-G4 plasmid was generated by ligating a PCR amplified rat GATA4 fragment (amino acids 191–445) into SalI-XbaI sites of the pBIND vector (Promega). Similarly, VP16 activation domain fused to PIAS1 was constructed by ligating PIAS1 fragment (amino acids 12 to 511) into BamHI sites of the activation domain vector, pACT (Promega). Sequences of all the primers used for plasmid construction are listed in Table S1.

### Site directed mutagenesis

Site directed mutagenesis of GATA4 and PIAS-1 was performed using the Quick-change II XL kit (Stratagene). The SUMO acceptor lysine 366 of GATA4 was mutated to arginine and the cysteine 350 within the catalytic RING domain of PIAS1 was mutated to serine. Sequences of mutagenic primers are listed in Table S1.

### Two-hybrid screening

Yeast two-hybrid screening was performed using the Matchmaker Gal4 two-hybrid system 3 (Clontech) according to manufacturer's instructions. The bait vector was constructed by subcloning a PCR generated rat GATA4 fragment (amino acids 191–445) in-frame with the Gal4 DNA binding domain in the yeast pGBKT7 expression vector. This GATA4 fragment has both zinc fingers and the C-terminal domain but lacks the N-terminal activation domains. The bait vector and human small intestinal cDNA library fused to Gal4 activation domain were transformed into the yeast strain AH109 and transformants were selected for growth on triple drop out media (−Trp1/−Leu2/−His3) and activation of α-galactosidase. Surviving colonies were streaked on quadruple dropout media (−Trp1/−Leu2/−His3/−Ade2) and the plasmid DNA from the clones that survived this second round of stringent screening were rescued, retransformed along with the bait into yeast strain AH109 to confirm interactions. Rescued plasmids were sequenced and the sequences were searched against GenBank entries to identify GATA4-interacting proteins.

### Cell culture and transfections

HCT116 colon cancer cells were purchased from ATCC and maintained in McCoy's 5A medium supplemented with 10% fetal bovine serum, 100 U/ml penicillin, 100 µg/ml streptomycin and 2 mM L-glutamine. Transient transfection experiments were performed by cotransfecting subconfluent cells with 0.2 µg of IFABP, SI and lactase promoter-luciferase vectors with 0.2 µg to 0.4 µg of GATA4 and PIAS-1 expression vectors. pCDNA3 empty vector was used to keep the total amount of transfected DNA constant.

### Chromatin immunoprecipitation (ChIP)

ChIP assays were performed on isolated mouse jejunal villus epithelium using a ChIP assay kit as described earlier [25]. Jejunal villus epithelium was isolated as described by Gu et al [26]. Briefly, jejunum from euthanized 6–8 week-old mice were opened and flushed several times using ice-cold PBS containing protease inhibitors to remove debris. The jejunum was cut into 1–2 cm segments, placed in ice-cold BSS buffer containing protease inhibitors, vortexed at maximum speed for 5 minutes and the supernatant containing mostly debris and a few villi was separated from the pellet by allowing the suspension to stand for 3 minutes. The pellet was suspended in fresh BSS buffer, vortexed for 5 minutes and passed through a 500 µ steel mesh (Spectrum Labs). The flow through containing the villi and a few crypts were pelleted, washed twice in HBSS and suspended in HBSS. ChIP assays were performed using a ChIP assay kit (Upstate/Millipore) using 5 µg of goat PIAS1 antibody (Santacruz Biotechnology) or nonimmune goat antibody (negative control) or acetyl histone H3 antibody (positive control). Chromatin immunoprecipitates were analyzed by semiquantitative PCR using primers corresponding to the promoter region (−306 and +24) or the exon 3 to exon 4 region (+3328 to +3981) of mouse IFABP gene. Sequences of primers used for ChIP assays are listed in supplementary data table 1.

### In vitro translation and GST pull-down assays

In vitro translations were performed using coupled rabbit reticulocyte in vitro transcription-translation system (Promega) according to manufacturer's instructions. GST pulldown experiments were performed as described earlier [5] using bacterially expressed, GST fused GATA4 and PIAS-1 proteins and ^35^ [S]-methionine labeled *in vitro* translated wild-type and mutated GATA4 and PIAS-1 proteins.

### Immunoprecipitation (IP) and western blotting (WB)

IP and WB experiments were done as described earlier [5], using lysates prepared from HCT116 cells transiently transfected with HA epitope tagged GATA4 and FLAG epitope tagged PIAS-1. While preparing lysates for in vivo sumoylation assays, N-ethyl maleimide was added to the lysis buffer to a final concentration of 20 mM. For immunoprecipitation of endogenous GATA4, 1 mg of lysates prepared from subconfluent IEC-6 cells were immunoprecipitated with 5 µg of goat GATA4 antibody (Santacruz Biotechnology) or non-immune goat control antibody. IPs were divided in to 2 and analyzed by blotting with rabbit SUMO-1 antibody (Cell Signaling) and mouse GATA4 antibody (Santacruz Biotechnology).

### Immunofluorescence

HCT 116 cells were plated in 6-well plates containing sterile coverslips and transfected with HA epitope tagged wild-type or K366R mutated GATA4. Thirty six hours posttransfection, cells were fixed in 2% paraformaldehyde and permeabilized with 0.2% triton X-100. Coverslips were washed in PBS and blocked with 5% horse serum for 1 hour at room temperature. Coverslips were incubated with 1∶500 diluted rabbit HA antibody in blocking buffer for 1 hour at room temperature, washed 5 times in PBS and incubated with secondary antibody (goat anti rabbit antibody) conjugated to Alexa 594. Coverslips were washed in PBS, mounted in DAPI-containing mounting media and photographed using BX50 microscope (Olympus, Center Valley, PA, USA) equipped with a CCD camera.

### Statistics

All numerical results are presented as mean ± SE. The statistical significance of differences was analyzed using Student's *t* test. *P*<0.05 was considered statistically significant.

## Results

### PIAS1 interacts with GATA4

**Figure 1:**
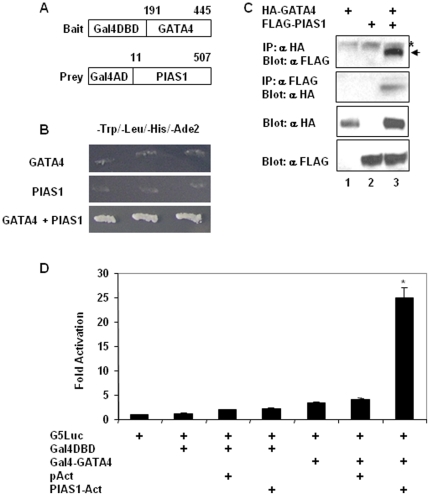
GATA4 and PIAS1 interact in yeast and mammalian cells. **Panel A.** The bait vector and the longest PIAS1 prey clone captured in the yeast two-hybrid screen are depicted diagrammatically. The numbers correspond to amino acids of the full length GATA4 and PIAS1 proteins. Abbreviations: Gal4DBD: Gal4 DNA binding domain; Gal4AD: Gal4 activation domain. **Panel B.** Yeast colonies transformed with rescued Gal4DBD-GATA4 (top panel) or Gal4AD-PIAS1 (middle panel) vectors or cotransformed with both (bottom panel) were streaked on quadruple dropout media. **Panel C.** HCT116 cells were transfected with HA epitope tagged GATA4 (lane 1) or FLAG epitope tagged PIAS1 (lane 2) or both (lane 3). Equal amounts of total protein lysates were immunoprecipitated with HA antibody and probed with FLAG antibody (top panel) or immunoprecipitated with FLAG antibody and probed with HA antibody (second panel). Third and fourth panels correspond respectively to western blots of input lysates with HA antibody and FLAG antibody. In the top panel, asterisk and arrow indicates nonspecific bands and the specific band, respectively. **Panel D.** Subconfluent HCT116 cells were transfected with Gal4 DNA binding site controlled minimal promoter luciferase reporter (G5Luc) along with vectors expressing Gal4 DNA binding domain (DBD) or Gal4 DBD fused to GATA4 or VP16 activation domain or VP16 activation domain fused to PIAS1 alone or in combinations as indicated. Lysates were assayed for luciferase activity 48 hours post-transfection and normalized to total protein. Fold activation over that of G5Luc activity, which was set as one was calculated. Results from 3 experiments done in triplicates are shown as mean±SEM. *p<0.05.

To identify proteins that interact with GATA4, we used a Gal4 based yeast two-hybrid screen in which GATA4 fused to the Gal4 DNA binding domain was used as a bait and an intestinal library fused to Gal4 activation domain was used as prey (Figure 1A). PIAS1 appeared multiple times in the screen and the longest PIAS1 clone had 496 amino acids (amino acids 11 to 507) that contained a partial N-terminal SAP domain and the entire RING finger domain. Physical association between GATA4 and PIAS1 in yeast was confirmed by growth of colonies on stringent quadruple drop out media (−Trp1/−Leu2/−His3/−Ade2) [Figure 1B] and activation of α-galactosidase (data not shown) when the rescued GATA4 and PIAS1 vectors were transformed together but not individually into yeast AH109 strain. To demonstrate that GATA4 and PIAS1 physically interact in mammalian cells, we performed coimmunoprecipitation experiments in HCT116 colon cancer cells by overexpressing HA epitope tagged GATA4 and FLAG epitope tagged PIAS1. As shown in the figure 1C, FLAGPIAS1 was detected in the immunoprecipitate of HAGATA4 and in converse experiments, HAGATA4 was detected in the immunoprecipitate of FLAGPIAS1 only when the 2 expression plasmids were coexpressed. We further confirmed the interaction between GATA4 and PIAS1 by using mammalian two-hybrid assays. Transfection of HCT116 cells with the GAL4 DNA binding domain fused GATA4 and the VP16 activation domain fused PIAS1 together but not individually resulted in a strong expression of the cotransfected GAL4 DNA binding site regulated luciferase reporter suggesting that GATA4 and PIAS1 interact in vivo (Figure 1D).

### C-terminal zinc finger domain and the adjacent basic region of GATA4 and the RING finger domain and the adjacent C-terminal region of PIAS1 mediate the physical interaction between GATA4 and PIAS1

**Figure 2:**
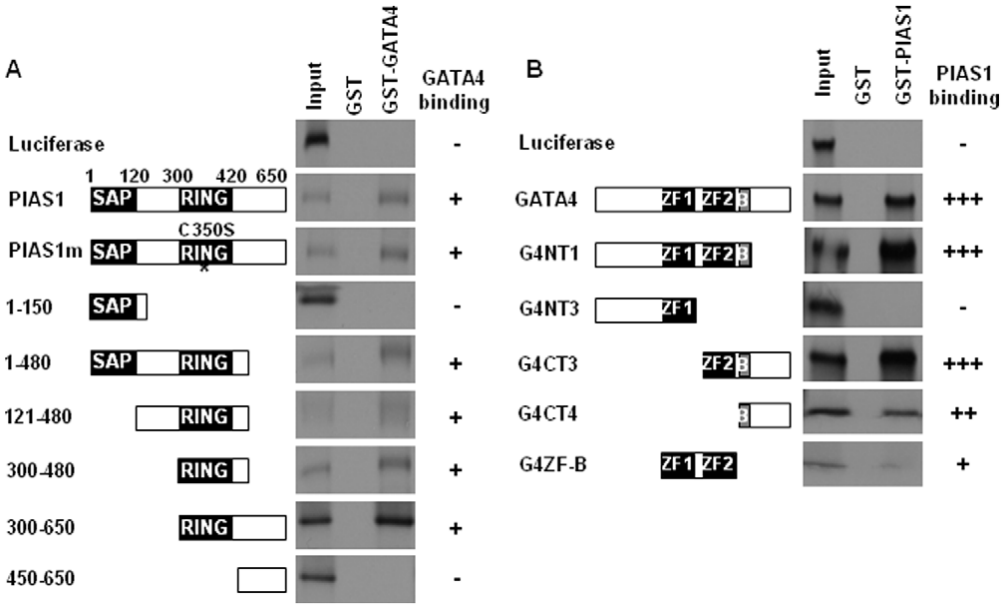
Second zinc finger and the adjacent basic region of GATA4 and the RING finger and the adjoining C-terminal sequences of PIAS1 mediate physical association. GST pull down experiments were performed using [^35^S] methionine labeled wild-type or indicated mutants of PIAS1 and purified GST fused GATA4 protein or GST protein immobilized on glutathione-sepharose beads (**panel A**). In **panel B**, converse experiments in which purified GST or GST fused PIAS1 protein immobilized on glutathione beads were used to pull down [^35^S] methionine labeled wild-type or indicated mutants of GATA4 protein is shown. The domains present in deletion mutants are diagrammatically indicated. The asterisk embedded in the RING domain of PIAS1m indicates that this protein carries a C350S mutation that inactivates the SUMO ligase activity.

We used GST pull-down assays to map the protein domains that mediate the physical interaction between GATA4 and PIAS1. Purified GST or GST-GATA4 fusion proteins were immobilized on glutathione-agarose beads and examined for their ability to interact with ^35^[S] methionine labeled in vitro translated wild-type and mutant PIAS1 proteins. As shown in figure 2A, both wild-type and SUMO ligase deficient C350S mutant of PIAS1 interacted with GST-GATA4 but not GST suggesting that the SUMO ligase activity is not required for the physical association between GATA4 and PIAS1. A mutant lacking either the N-terminal 300 amino acids or the C-terminal 170 amino acids (PIAS 1–480) bound GATA4 suggesting that the N-terminal 300 amino acids and the C-terminal 170 amino acids of PIAS1 are dispensable for GATA4 binding. Confirming this observation, the N-terminal 150 amino acid peptide (PIAS 1–150) containing the SAP domain and a 200 amino acid C-terminal peptide (PIAS 450–650) failed to interact with GATA4. A mutant containing the centrally located RING finger domain and lacking the N-terminal 121 amino acids and the C-terminal 170 amino acids interacted with GATA4 suggesting that the RING finger domain may be involved in the interaction of PIAS1 with GATA4. This notion was confirmed by demonstrating that the RING finger domain and a small adjoining C-terminal region of 60 amino acids encoded by PIAS 300–480 are sufficient for GATA4 binding (Figure 2A). The SUMO ligase activity of PIAS1 was not required for interaction because a mutant PIAS1 with C350S mutation that affects the SUMO ligase activity was comparable to wild-type PIAS1 in binding to GATA4 (Figure 2A).

A reciprocal approach in which GST-PIAS1 fusion protein immobilized on glutathione agarose beads was used to pull down ^35^[S]-methionine labeled, in vitro translated wild-type and mutant GATA4 proteins to map the domains of GATA4 that mediate its interaction with PIAS1 (Figure 2B). While wild-type GATA4 bound to PIAS1, mutant G4NT3 which has a deletion of the C-terminal zinc finger domain and the adjoining basic region and the C-terminal activation domain failed to bind PIAS1 suggesting that the PIAS1 interacting region is located within these C-terminal regions of GATA4. In agreement with this data, GATA4 peptide consisting of the C-terminal zinc finger, the adjoining basic region and the C-terminal activation domain (G4CT3) was sufficient for interaction with PIAS1. A deletion of the C-terminal activation domain (amino acids 335–440 in the construct NT1) did not affect interaction of GATA4 with PIAS1 suggesting that the C-terminal zinc finger region and the adjoining basic region may mediate interaction between GATA4 and PIAS1. Peptides consisting of solely of the zinc fingers (G4ZF-B) or the basic region and the C-terminal activation domain (G4CT4) bound to PIAS1 weakly suggesting that both the second zinc finger and the adjoining basic region together are required for strong binding to PIAS1.

### PIAS1 is recruited to GATA4 target gene promoter

**Figure 3:**
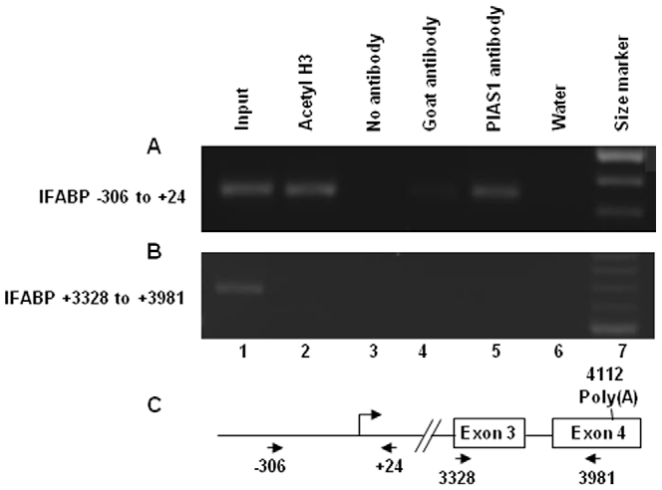
PIAS1 binds to IFABP promoter in isolated murine villus epithelial cells. Cross-linked chromatin isolated from murine villus epithelial cells were sheared and immunoprecipitated with acetylated histone H3 antibody (lane 2), no antibody (lane 3), nonimmune goat antibody (lane 4) and PIAS1 antibody (lane 5). The chromatin immunoprecipitates were purified, reverse cross-linked and analyzed by semiquantitative PCR with primers corresponding to IFABP promoter (**panel A**) or primers from 3′ region of the IFABP gene (**panel B**). Lane 1 is input control (5%). Lane 6 is water control for used during the PCR reaction. Size marker was run in lane 7. In **panel C**, the nucleotide coordinates of the primers with respect to IFABP genomic clone (GenBank accession # M65033) is shown. Cap site, exons and the poly(A) signal are indicated.

We examined whether the physical association of PIAS1 with GATA4 leads to recruitment of PIAS1 to IFABP, a GATA4 target gene, using a ChIP assay. Formaldehyde crosslinked chromatin isolated from mouse jejunal villus epithelium was sheared, immunoprecipitated with PIAS1 antibody or a nonimmune goat antibody (negative control) or acetylated histone H3 (positive control) and analyzed by PCR using primers that span the IFABP promoter. As shown in the figure 3A, DNA corresponding to IFABP promoter was immunoprecipitated by PIAS1 antibody demonstrating that PIAS1 is recruited to IFABP promoter. An exon 3 to exon 4 region of the IFABP was not precipitated by PIAS1 antibody indicating that association of PIAS1 with DNA bound GATA4 may be required for recruitment of PIAS1 to IFABP chromatin (Figure 3B).

### PIAS1 differentially coactivates GATA4 target gene promoters

**Figure 4:**
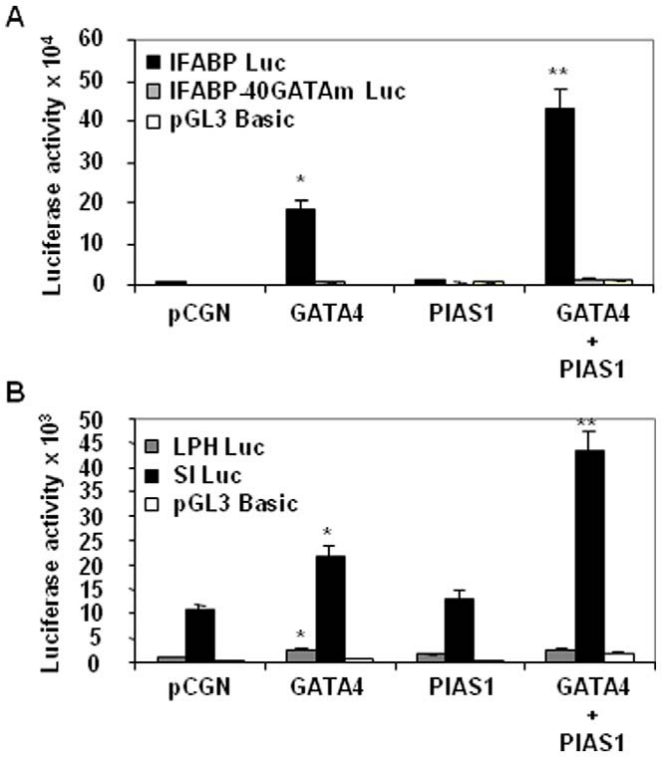
GATA4 and PIAS1 selectively coactivate GATA4 target promoters. Subconfluent HCT116 cells were transfected with pCGN empty vector or GATA4 or PIAS1 or GATA4 and PIAS1 together along with IFABP promoter or IFABP promoter with mutated GATA4 binding site or pGL3 basic luciferase reporters (**panel A**). In **panel B**, LPH promoter and SI promoter luciferase reporters were used. Lysates were assayed for luciferase activity 48 hours post-transfection. Results from 3 experiments done in triplicates are shown as mean±SEM. *p<0.05 for GATA4 transfected cells compared with pCGN transfected cells. **p<0.05 for GATA4 and PIAS1 cotransfected cells compared with GATA4 transfected cells.

Since PIAS1 interacted with GATA4 and recruited to the IFABP promoter, a GATA4 target, we examined if PIAS1 modulates the transcriptional activity of GATA4. As shown in the figure 4A, PIAS1 strongly enhanced the activation of IFABP promoter by GATA4 demonstrating that PIAS1 is a coactivator of GATA4. This coactivation was dependent on GATA4 binding to DNA because a mutation at the −40 GATA site that we have shown previously to mediate the activation of the promoter by GATA4 [5], abolished synergistic activation by GATA4 and PIAS1. We further examined if PIAS1 coactivates other GATA4 target promoters such as, LPH and SI. In HCT116 cells, the basal activity of LPH promoter was lower compared with that of SI promoter. Despite the differences in the basal promoter activity, both promoters were activated approximately 2-fold by GATA4. Interestingly, the activity of SI promoter but not the LPH promoter was enhanced by PIAS1 (Figure 4B). Together these results suggest that PIAS1 coactivates a subset of GATA4 target gene promoters and helps differentiate among GATA4 target promoters.

### Mapping of GATA4 and PIAS1 domains required for IFABP coactivation

**Figure 5:**
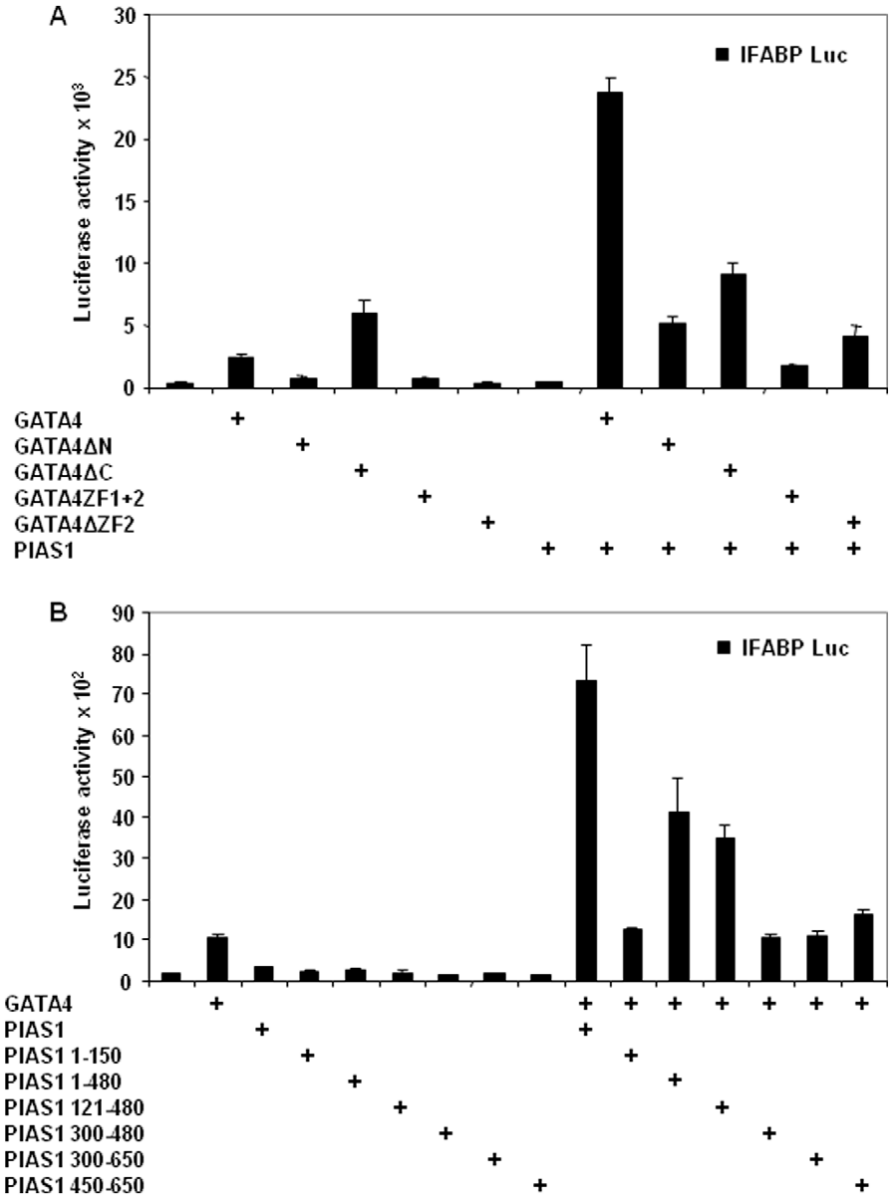
Mapping of GATA4 and PIAS1 domains required for IFABP coactivation. Transient cotransfections were performed in subconfluent HCT116 cells using wild-type and deletion mutants of GATA4 and wild-type PIAS1 (**panel A**). In **panel B**, wild-type and deletion mutants of PIAS1 and wild-type GATA4 were used for cotransfections. Lysates were assayed for luciferase activity 48 hours post-transfection. Results from 3 experiments done in triplicates are shown as mean±SEM.

We determined the domains of GATA4 required for IFABP coactivation by transfecting GATA4 deletion mutants along with PIAS1 and IFABP promoter-luciferase reporter into HCT116 cells. Deletion of the N-terminal activation domains abolished synergism between GATA4 and PIAS1 (Figure 5A). As noted earlier [5], GATA4 with a deletion of the C-terminal conformation-dependent activation domain activated IFABP promoter more strongly than the wild-type GATA4, yet this mutant failed to synergistically activate IFABP. A mutant consisting of only the N- and C-terminal zinc fingers was not sufficient for coactivation. Deletion of the DNA binding C-terminal zinc finger abolished synergism between GATA4 and PIAS1. Together these results suggest that the N- and C-terminal activation domains as well as DNA binding by GATA4 are required for coactivation of IFABP by GATA4 and PIAS1.

To map the domains of PIAS1 required for coactivation with GATA4, we used PIAS1 deletion mutants. As shown in the figure 5B, the N-terminal SAP domain and the adjacent 30 amino acids did not support coactivation (PIAS1 1–150). The coactivation was reduced by 50% for mutants that lacked either the C-terminal 170 amino acids (PIAS1 1–480) or both the C-terminal 170 amino acids and the N-terminal 120 amino acids (PIAS1 121–480). A common feature of these 2 mutants is the RING domain and the adjacent N-terminal 180 amino acids. Deletion of these 180 amino acids in the construct PIAS1 300–450, which has intact RING domain, abolished coactivation suggesting that these 180 amino acids are required for coactivation. Coactivation was not restored for the construct PIAS1 300–650 which has the RING domain and the entire C-terminal domain. The C-terminal domain (PIAS1 450–650) by itself was not sufficient for coactivation. Together, these results indicate that while the centrally located RING domain and the adjacent N-terminal 180 amino acids are sufficient for coactivation, maximal coactivation requires additional domains located at the N- and C- terminus of PIAS1.

### GATA4 is sumoylated and PIAS1 promotes GATA4 sumoylation

**Figure 6:**
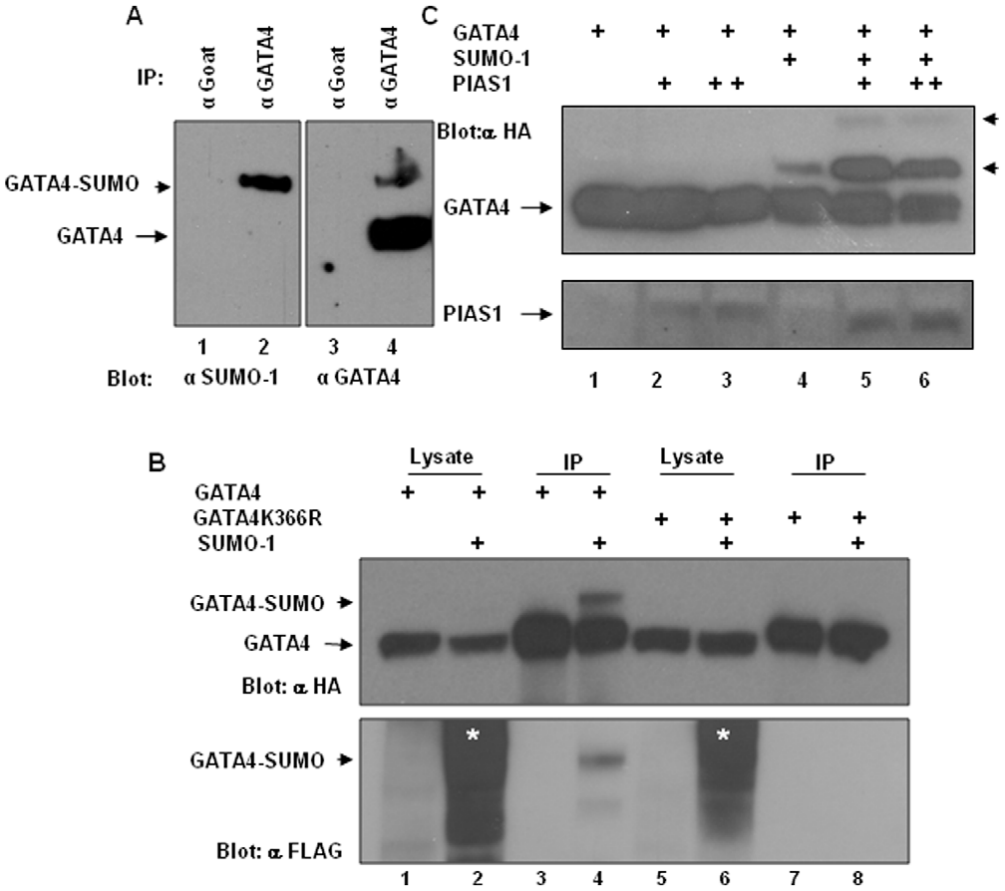
GATA4 is sumoylated and PIAS1 promotes GATA4 sumoylation. **Panel A.** IEC-6 cell lysates prepared in the presence of 20 mM N-ethyl maleimide were immunoprecipitated with nonimmune goat or goat GATA4 antibody. IPs were divided in to 2 and analyzed by blotting with rabbit SUMO-1 antibody (left panel) and mouse GATA4 antibody (right panel). **Panel B.** Top panel: HCT116 cells were transfected with HA epitope tagged wild-type or K366R mutated GATA4 with or without FLAG epitope tagged SUMO-1. Lysates were prepared in buffer containing 20 mM N-ethyl maleimide and equal amounts of lysates were immunoprecipitated with mouse HA antibody. Immunoprecipitates (lanes 3,4,7,8) and corresponding input controls (lanes 1,2,5,6) were analyzed by western blotting with rabbit HA antibody. Nonsumoylated GATA4 and sumoylated GATA4 are indicated, respectively, by arrow and arrowhead. Bottom panel: The blot shown in the top panel was stripped and reprobed with rabbit FLAG antibody. The dark smear in the input controls representing cellular sumoylated proteins is indicated by asterisk. Arrowhead points to the sumoylated GATA4. **Panel C.** Top panel: HA epitope tagged GATA4 was transfected with SUMO-1 and PIAS1 as indicated. Cell lysates were prepared and immunoprecipitated with mouse HA antibody as indicated in panel A and analyzed by western blotting with rabbit HA antibody. Nonsumoylated GATA4 and sumoylated GATA4 are indicated, respectively, by arrow and arrowhead. Bottom panel: The blot shown in the top panel was stripped and reprobed with goat PIAS1 antibody.

PIAS1 is a SUMO E3 ligase. Since GATA4 physically interacted with PIAS1, we examined if GATA4 is sumoylated in intestinal epithelial cells and if PIAS1 promotes GATA4 sumoylation. To determine if the endogenous GATA4 is sumoylated in intestinal epithelial cells, lysates prepared from the rat jejunal crypt-derived IEC-6 cells were IPd with goat GATA4 antibody and the IPs were analyzed by probing with mouse GATA4 and rabbit SUMO-1 antibodies. GATA4 antibody recognized a major 50 kD band (the expected size of GATA4) and a minor slow migrating band of approximately 70 kD. The SUMO-1 antibody recognized this 70 kD band suggesting that this band corresponds to SUMO modified GATA4 (Figure 6A). Additionally, we cotransfected HCT116 colon epithelial cells with HA epitope tagged GATA4 and FLAG epitope tagged SUMO-1 and the cell lysates were IPd with mouse HA antibody and analyzed by western blotting with a rabbit HA antibody. As shown in the figure 6B, top panel, cotransfection of HA epitope tagged GATA4 and FLAG epitope tagged SUMO-1 resulted in the appearance of a slow migrating GATA4 band. This slow migrating GATA4 band reacted with the FLAG antibody suggesting that the slow migrating GATA4 band corresponds to SUMO modified GATA4 (Figure 6B, lower panel). Further, this slow migrating GATA4 band was absent when a GATA4K366R, which has the SUMO acceptor lysine 366 mutated to arginine, was used for transfection instead of the wild-type GATA4. These results are consistent with a previous study that demonstrated that GATA4 is sumoylated on K366 in HeLa cervical carcinoma cells and cardiac myocytes [23]. Since PIAS1 is a SUMO E3 ligase for various sumoylated proteins including GATA4, and physically associated with GATA4 via the RING finger domain, we examined whether PIAS1 promotes GATA4 sumoylation in intestinal epithelial cells by cotransfecting wild-type PIAS1 or SUMO ligase deficient, C350S mutated PIAS1 along with HA epitope tagged GATA4 and SUMO-1 into HCT116 cells. As shown in the figure 6B, top panel, PIAS1 promoted GATA4 sumoylation. PIAS1C350S mutant PIAS1 failed to do so (data not shown).

### Sumoylation is not required for nuclear localization of GATA4 in intestinal epithelial cells

**Figure 7:**
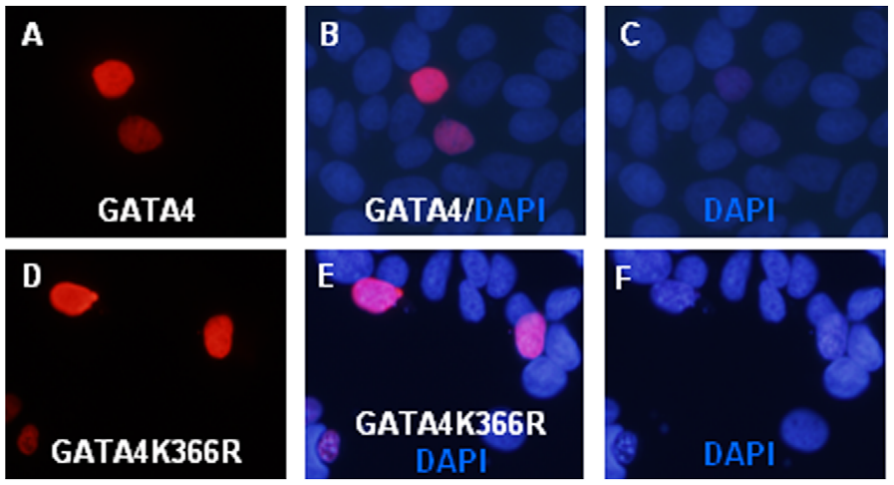
Sumoylation is not required for nuclear localization of GATA4. HCT116 cells plated on coverslips were transfected with HA epitope tagged GATA4 (panels A,B,C) or K366R mutated GATA4 (panels D,E,F). Cells were fixed, permeabilized and stained with rabbit HA antibody and Alexa 594 conjugated anti rabbit secondary antibody. Coverslips were mounted with DAPI-containing mounting media and analyzed for immunofluorescence.

Previously it was shown that sumoylation is an important determinant of GATA4 nuclear localization [23]. Abolishing GATA4 sumoylation by mutating the SUMO acceptor K365 or interfering with GATA4 sumoylation by knocking down the obligatory SUMO E2 conjugase, Ubc9, prevented GATA4 nuclear localization in HeLa cervical carcinoma cells. We examined whether GATA4 nuclear localization in intestinal epithelial cells is also dependent on sumoylation by immunofluorescence analysis of HCT116 and IEC-6 cells transfected with HA tagged wild-type or K366R mutated GATA4. In both HCT116 cells (Figure 7) and IEC-6 cells (data not shown) K366R mutant GATA4 was localized to the nucleus, similar to wild-type GATA4 suggesting that sumoylation is dispensable for GATA4 nuclear localization in intestinal epithelial cells. Further, cycloheximide chase experiments indicated that both wild-type and K366R mutated GATA4 have similar protein half lives suggesting that sumoylation does not affect GATA4 protein stability (data not shown).

### Neither GATA4 sumoylation nor PIAS1 SUMO ligase activity is required for coactivation of IFABP promoter

**Figure 8:**
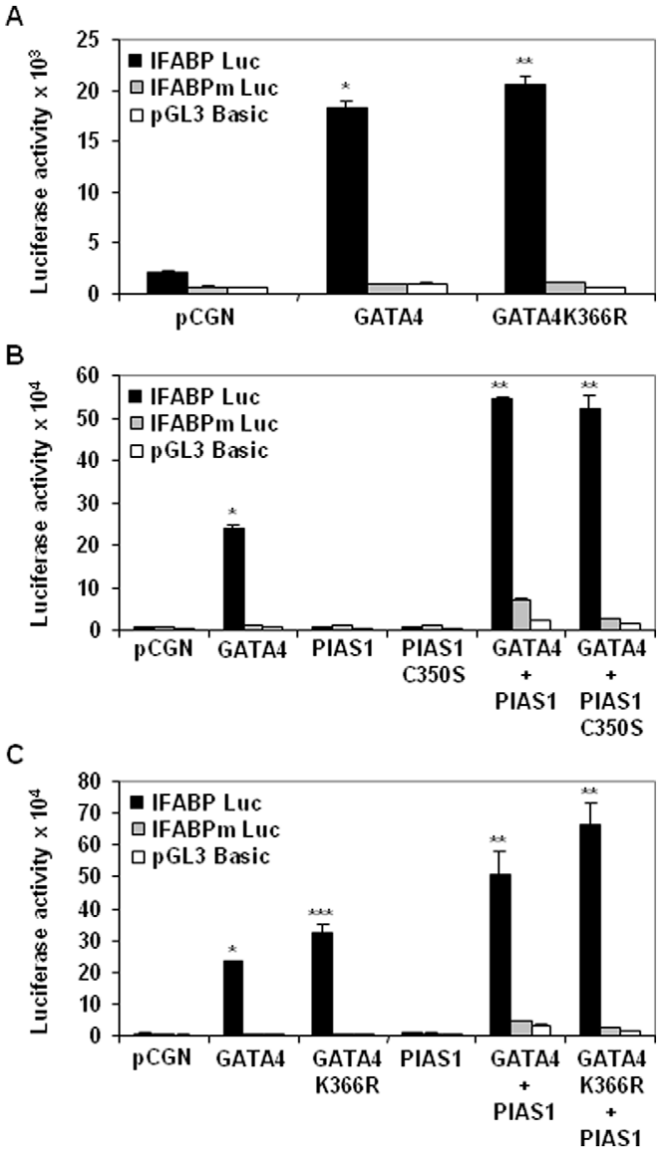
GATA4 sumoylation and PIAS1 SUMO ligase activity is required for coactivation of IFABP promoter. **Panel A.** Subconfluent HCT116 cells were transfected with pCGN empty vector or wild-type GATA4 or K366R mutated GATA4 along with IFABP promoter or IFABP promoter with mutated GATA4 binding site or pGL3 basic luciferase reporters. Lysates were assayed for luciferase activity 48 hours post-transfection. Results from 3 experiments done in triplicates are shown as mean±SEM. *p<0.05 for GATA4 transfected cells compared with pCGN transfected cells. **p<0.05 for GATA4K366R transfected cells compared with GATA4 transfected cells. **Panel B.** Transient transfections in HCT116 cells were carried out as indicated in panel A with GATA4 or PIAS1 or C350S mutated PIAS1 individually or in combination as shown. *p<0.05 for GATA4 transfected cells compared with pCGN transfected cells. **p<0.05 for GATA4 and PIAS1/PIAS1C350S cotransfected cells compared with GATA4 transfected cells. **Panel C.** Transfections were performed in HCT116 cells as indicated in panel A using GATA4 or K366R mutants with or without PIAS1. *p<0.05 for GATA4 transfected cells compared with pCGN transfected cells. **p<0.05 for GATA4/PIAS1 and GATA4K366R/PIAS1 cotransfected cells compared with GATA4 and GATA4K366R transfected cells. ***p<0.05 for GATA4K366R transfected cells compared with GATA4 transfected cells.

SUMO modification of GATA4 is essential for transactivation of cardiac tissue-restricted GATA4 target genes and GATA4 induced cardiomyogenic differentiation of 10T1/2 fibroblasts [23]. To examine whether GATA4 sumoylation is also important for the transactivation of GI expressed promoters in intestinal epithelial cells we cotransfected HCT116 cells with IFABP promoter-luciferase reporter along with wild-type or nonsumoylatable K365R mutated GATA4. As shown in the figure 8A and 8C, GATA4K366R activated the IFABP promoter more strongly than the wild-type GATA4 indicating that transactivation of GI promoter by GATA4 is independent of sumoylation. Since SUMO modification on GATA4 was not required for transactivation, we analyzed whether the SUMO ligase activity of PIAS1 was required for coactivation of IFABP promoter. SUMO ligase deficient PIAS1 C350S mutant was comparable to wild-type PIAS1 for IFABP promoter coactivation (Figure 8B). Further, the C350S mutated PIAS1 synergized with non sumoylatable K366R mutated GATA4 (Figure 8C). Together these results show that neither GATA4 sumoylation nor the PIAS1 SUMO ligase activity is required coactivation of IFABP promoter.

## Discussion

GATA4 regulation of cell-type-specific gene expression is achieved through the combinatorial interaction of GATA4 with various ubiquitous and cell-type-enriched transcriptional regulators and coregulators [1]. Our study has determined that PIAS1 is an intestine-expressed GATA4 partner protein.

GATA4 and PIAS1 physically associated with each other as demonstrated by interaction assays performed in yeast or in mammalian cells or in vitro. The physical association involved the second zinc finger and the adjacent basic region of GATA4 and the RING finger and the adjoining C-terminal sequences of PIAS1. The physical association enabled GATA4 and PIAS1 to synergistically activate IFABP promoter. Since this synergism was dependent on GATA4 binding to DNA, the results suggested that PIAS1 was recruited to IFABP promoter via its interaction with GATA4. In addition to IFABP, SI, a known GATA4 dependent promoter was also synergistically activated. However, not all GATA4-dependent promoters were coactivated by GATA4 and PIAS1. For example, LPH promoter was activated by GATA4 but failed to be coactivated by GATA4 and PIAS1 suggesting that the selective recruitment of PIAS1 to GATA4-dependent promoters may determine the expression levels of specific promoters. Considering that PIAS1 can bind to chromatin through its SAP domain and localize to nuclear speckles often considered to be sites of active chromatin [27], it is possible that in the context of chromatin under in vivo conditions, PIAS1 may associate with chromatin around specific GATA4 target genes and subsequently recruit GATA4.

There are many factors known to interact with GATA4 and regulate GATA4 transcriptional activity [28]. A majority of these interacting proteins such as, SRF [29], Nkx2-5 [30], [31], TBX5 [32], Hand2 [33], Sp1 [34], SF1 [35], HNF1 alpha [9], Smads [5], [36] and Fog2 [19], may work as part of transcriptional complexes involving GATA4. Several other partners of GATA4 such as, p300 [37], HDAC2 [38], Erk-1/2 [39], p38 MAP kinase [40], protein kinase A [41], and protein kinase C [42] act by both associating with and inducing or eliminating posttranslational modifications on GATA4 protein. PIAS1 belongs to the latter group that both interacts with and modifies GATA4. Interestingly, GATA4 modification was not required for GATA4 transcriptional activity and preventing GATA4 modification did not affect nuclear localization of GATA4. Further, PIAS1 SUMO ligase activity was not required for coactivation. These observations contrast with that of Wang et al [23], who showed that sumoylation regulates nuclear localization and transcriptional activation functions of GATA4 and the SUMO ligase activity of PIAS1 is important for synergy between GATA4 and PIAS1. This discrepancy could be due to the differences in the gene promoters analyzed and the cell types used. These contrasting observations point to a fundamentally different mechanism by which SUMO pathway components regulate GATA4 activity in cardiac and GI cell types resulting in distinct outcomes. There is also precedence that SUMO ligases can regulate gene expression independently of their SUMO ligase activities [43]. For example, SF1, a GATA4 partner protein, is sumoylated and its sumoylation is promoted by PIAS1 and PIAS3 [44]. Sumoylation regulates the transcriptional activities of SF-1. PIAS1 can enhance transcriptional activity of SF-1 on select SF-1 target promoters but not all SF-1 target promoters and independent of SUMO ligase activity [45].

Although sumoylation *per se* is not required for GATA4 transactivation of IFABP promoter, SUMO may have a role in regulating GATA4 activity. We have found that both wild-type and nonsumoylatable K366R mutated GATA4 synergizes with SUMO-1 to activate IFABP promoter (unpublished observations). This synergism may be mediated via non covalent interactions between GATA4 and SUMO-1. SUMO-interaction motif (SIM), a loose consensus consisting of a core of ψψ×ψ or ψ×ψψ (where ψ is a hydrophobic amino acid and x is any amino acid) often with a cluster of acidic amino acids at the C-terminus mediates such non covalent interactions [43], [46]. Several such loose consensus sequences (LDMFDD and LYMKL) are present in GATA4. Non covalent interactions between such potential SIMs in GATA4 and SUMO proteins and other sumoylated GATA4 partner proteins may be important for regulation of GATA4 mediated gene expression in GI epithelial cells.

Transcriptional activity of several factors has been shown to be limited by a stretch of amino acids of consensus sequence “P-X_(0,4)_-I/V-K-Q/T/S/L/E/P-E-X_(0,3)_-P”, termed synergy control (SC) motif, the deletion of or mutations in which enhances transcriptional activity by allowing transcriptional regulators to work in synergy [47]. GATA4 has such a motif in the C-terminus and deletion of the C-terminus that consists of this motif increased transcriptional activity of GATA4 (Figure 5A, labeled GATA4ΔC). Subsequently, the core of the SC motif, “ΨxKE” was determined to be a consensus sequence for sumoylation [48]. In GATA4 a mutation of K366 that disrupts the SC consensus/sumoylation sequence increased transcriptional activity of GATA4 and enhanced its ability to coactivate with PIAS1 (Figure 8). The activity of this motif can be modulated by sumoylation, which is dynamic and readily reversible. Considering that SUMO moiety recruits several corepressor activities that can epigenetically remodel chromatin [49]–[52], GATA4 sumoylation site provides a platform dependent on which sumoylated GATA4 may repress gene expression while nonsumoylated GATA4 will serve as an activator of gene expression. Several transcriptional regulators such as, Elk-1, Sp3 and p300, serve as both activators and repressors, depending on their sumoylation status [51]–[53]. Existence of a GATA4 sumoylation switch-dependent mutually exclusive gene programs may help explain the role of GATA4 in establishing and maintaining jejunal identity. Intestine-specific inactivation of GATA4 has revealed that GATA4 is essential for activating a jejunum-specific gene program while simultaneously repressing a ileum-specific gene program [2], [3]. The repression of ileal gene program in jejunum was dependent on the interaction of GATA4 with GATA4 cofactor, Fog1 [18], a protein that binds repressor complexes in a sumoylation-dependent manner and differentially regulates erythroid differentiation program of GATA1 [54]. These observations add to the credence that nonsumoylated GATA4 may promote jejunum-specific gene expression while sumoylated GATA4, capable assembling corepressor complexes, may repress ileum-specific gene program.

In GI epithelial cells sumoylation is an important posttranslational modification essential for determination and differentiation of intestinal epithelial cell types. A recent study showed that an inducible ablation of Ubc9, the only E2 SUMO conjugase activity essential for sumoylation of all SUMO substrates, affected enterocyte differentiation, shortened villus height and increased the villus goblet cell population [55]. This phenotype partially resembles the intestine-specific GATA4, GATA6 and compound GATA4/GATA6 ablation phenotypes characterized by shortened villus height, reduced crypt cell proliferation, increased goblet cell numbers and impaired jejunal enterocyte differentiation program [2], [3], [56]. The similarity in the intestinal phenotype between Ubc9, which eliminates sumoylation globally, and GATA4/GATA6 loss of function suggests that SUMO modification of GATA4 and other sumoylatable intestinal transcriptional regulators may be important for their intestinal epithelial cell-type determination and differentiation activities.

## Supporting Information

Table S1 **List and sequence of primers used for plasmid constructions.**
(DOC)
