# Correction: PIAS1 Is a GATA4 SUMO Ligase That Regulates GATA4-Dependent Intestinal Promoters Independent of SUMO Ligase Activity and GATA4 Sumoylation

**DOI:** 10.1371/annotation/e56fbda3-1898-4a36-bf93-d33d5a8b2d2e

**Published:** 2012-09-26

**Authors:** Narasimhaswamy S. Belaguli, Mao Zhang, Andrés Hernández-Garcia, David H. Berger

The third author's name is misspelled. The correct name is: Andrés Hernández-García.

The correct citation is: Belaguli NS, Zhang M, Hernández-García A, Berger DH (2012) PIAS1 Is a GATA4 SUMO Ligase That Regulates GATA4-Dependent Intestinal Promoters Independent of SUMO Ligase Activity and GATA4 Sumoylation. PLoS ONE 7(4): e35717. doi:10.1371/journal.pone.0035717 

