# Supplementary material for: PIAS1 Is a GATA4 SUMO Ligase That Regulates GATA4-Dependent Intestinal Promoters Independent of SUMO Ligase Activity and GATA4 Sumoylation
Source: PLoS One. 2012 Apr 23;7(4):e35717. doi: 10.1371/journal.pone.0035717 (PMC3334497; doi:10.1371/journal.pone.0035717)
Supplement: Table S1 — List and sequence of primers used for plasmid constructions. (DOC) [file pone.0035717.s001.doc]

**Table S1.**

**List and sequence of primers used for plasmid constructions**

GATA4 191(T) 5’-ccgctcgagGCCTACCTGGCCGGGCCAACCCT-5’

GATA4 445(B) 5’-tgctctagaTTACGCGGTGATTATGTCCCC-3’

Human LPH -399(T) 5’-cggggtaccCCGCTTGGCTCCTGCAACCTCCGCCT-3’

Huma LPH +14(B) 5’-ggaagatctTCCGCCAAGACAGCTCCATTTTCTAGGA-3’

Human SI -342(T) 5’-cgacgcgtCCAGTGAGGATCTAAATTGCA-3’

Human SI +30 (B) 5’-ccgctcgagCCAGACTTGGATAAGGCTGCC-3’

PIAS1 (T) 5’-ggcggatccgcATGGCGGACAGTGCGGAACTA-3’

PIAS1 12(T) 5’-cgcggatccTTATGAGCCTTAGAGTTTCTG-3’

PIAS1 121(T) 5’-ggcggatccgcATGGAACTCCCACATCTCACGTCA-3’

PIAS1 300(T) 5’-ggcggatccgcATGGGAATAAGGAATCCGGATCAT-3’

PIAS1 450(T) 5’-ggcggatcc GCATGTCAAATAAAAACAAGAAAGTCGAG-3’

PIAS1 150(B) 5’-cccaagcttTCACAGTTCATCCAACAGGCTATAGAA-3’

PIAS1 480(B) 5’-cccaagcttTCAGGTCCTCTTGGCAGGGGGTTC-3’

PIAS1 511(B) 5’-cgcggatccTCAAAGGCTTGGGGTGCGGGAC-3’

PIAS1 650(B) 5’-cccaagcttTCAGTCCAATGAGATAATGTCTGG-3’

Nucleotides in lower case letters were designed into oligonucleotides to facilitate cloning and were not part of the indicated genes. Unerlined sequences correspond to the following restriction enzyme sites: KpnI in human LPH -399 primer; BglII in human +14 primer; MluI in human SI -342 primer; XhoI in +30 human SI and GATA4 191(T) primers; HindIII in PIAS1 150(B), PIAS1 650(B), PIAS 480(B) primers; BamHI in PIAS1 (T), PIAS1 12(T), PIAS1 121(T), PIAS1 300(T), PIAS1 450(T), PIAS1 511(B) primers and XbaI in GATA4 445(B) primer. Abbreviation (T) and (B) in brackets denote the strand of the oligonucleotide.

**List and sequence of primers used for site directed mutagenesis**

GATA4 K366R (T) 5’-GCGCCCCATCAgGACAGAGCCCG-3’

PIAS1 C350S (T) 5’-CTGTCGGGCACTTACCaGCTCCCACCTTCA-3’

Only the top strand sequence is shown for these mutagenic primers. The bottom strand is the complement of the top strand. Mutated nucleotides are shown in lower case.

**List and sequence of primers used for ChIP analysis**

Mouse IFABP -306(T) 5’-CTTAGAACTGGCTGCCTCTG-3’

Mouse IFABP +24(B) 5’-TGTGTGTCTCTAGGAAAGCA-3’

Mouse IFABP +4295(T) 5’-CTGGACCATTGAGGGAAATA-3’

Mouse IFABP +4949(B) 5’-CCTGGCATTAGCATGATGCA-3’

Abbreviation (T) and (B) in brackets denote the strand of the oligonucleotide.
